# *Mig-6* Plays a Critical Role in the Regulation of Cholesterol Homeostasis and Bile Acid Synthesis

**DOI:** 10.1371/journal.pone.0042915

**Published:** 2012-08-17

**Authors:** Bon Jeong Ku, Tae Hoon Kim, Jae Hee Lee, Eric D. Buras, Lisa D. White, Robert D. Stevens, Olga R. Ilkayeva, James R. Bain, Christopher B. Newgard, Francesco J. DeMayo, Jae-Wook Jeong

**Affiliations:** 1 Department of Molecular and Cellular Biology, Baylor College of Medicine, Houston, Texas, United States of America; 2 Department of Internal Medicine, Chungnam National University School of Medicine, Daejeon, Korea; 3 Department of Obstetrics, Gynecology & Reproductive Biology, Michigan State University, Grand Rapids, Michigan, United States of America; 4 Microarray Core Facility, Department of Molecular & Human Genetics, Baylor College of Medicine, Houston, Texas, United States of America; 5 Sarah W. Stedman Nutrition and Metabolism Center, Department of Pharmacology and Cancer Biology and Department of Medicine, Duke University Medical Center, Durham, North Carolina, United States of America; Clermont Université, France

## Abstract

The disruption of cholesterol homeostasis leads to an increase in cholesterol levels which results in the development of cardiovascular disease. Mitogen Inducible Gene 6 (*Mig-6*) is an immediate early response gene that can be induced by various mitogens, stresses, and hormones. To identify the metabolic role of *Mig-6* in the liver, we conditionally ablated *Mig-6* in the liver using the Albumin-Cre mouse model (*Alb^cre/+^Mig-6^f/f^*; *Mig-6^d/d^*). *Mig-6^d/d^* mice exhibit hepatomegaly and fatty liver. Serum levels of total, LDL, and HDL cholesterol and hepatic lipid were significantly increased in the *Mig-6^d/d^* mice. The daily excretion of fecal bile acids was significantly decreased in the *Mig-6^d/d^* mice. DNA microarray analysis of mRNA isolated from the livers of these mice showed alterations in genes that regulate lipid metabolism, bile acid, and cholesterol synthesis, while the expression of genes that regulate biliary excretion of bile acid and triglyceride synthesis showed no difference in the *Mig-6^d/d^* mice compared to *Mig-6^f/f^* controls. These results indicate that *Mig-6* plays an important role in cholesterol homeostasis and bile acid synthesis. Mice with liver specific conditional ablation of *Mig-6* develop hepatomegaly and increased intrahepatic lipid and provide a novel model system to investigate the genetic and molecular events involved in the regulation of cholesterol homeostasis and bile acid synthesis. Defining the molecular mechanisms by which *Mig-6* regulates cholesterol homeostasis will provide new insights into the development of more effective ways for the treatment and prevention of cardiovascular disease.

## Introduction

Cardiovascular disease is one of the leading causes of death in the world [1]. Hypercholesterolemia has been recognized as a major risk factor contributing to the development of cardiovascular disease [1], [2], [3]. Defining the molecular mechanisms regulating cholesterol homeostasis will lead to more effective ways of treating and preventing cardiovascular disease [4]. Cholesterol is essential for life and plays an important role in mammalian cells. Under normal physiological conditions, cholesterol input is equal to its output [5]. Multiple, tightly coordinated processes are involved in the maintenance of cholesterol homeostasis. Disruption of these processes leads to an increase in cholesterol levels and eventually causes the development of cardiovascular disease [3], [4], [5].

Bile acids are amphipathic molecules synthesized from cholesterol in the liver [6]. The major functions of bile acids are cholesterol elimination, lipid transport in the form of mixed micelles, and stimulation of biliary phospholipid secretion [7]. In order to accomplish these functions, bile acids are distributed via continuous enterohepatic circulation [6], [7]. Disturbances in bile acid synthesis, transport, and circulation cause several metabolic diseases, such as Zellweger syndrome [7]. The identification of bile acid homeostasis regulators is critical in understanding how these processes are altered in disease states. Here, we report that Mitogen Inducible Gene 6, *Mig-6*, is a critical regulator of bile homeostasis.

Previously, we have identified Mitogen Inducible Gene 6, *Mig-6* (Effri1, RALT, or gene 33), as a target of progesterone receptor and Steroid Receptor Coactivator 1 (SRC-1) action in the uterus [8], [9]. *Mig-6* is an immediate early response gene that can be induced by various mitogens, stresses, and hormones [10], [11], [12], [13]. *Mig-6* is an adaptor molecule containing a CRIB domain, a src homology 3 (SH3) binding domain, a 14-3-3 binding domain, and an EGFR binding domain [10], [14], [15]. A decreased expression of *Mig-6* is observed in human breast carcinoma which correlates with reduced overall survival of breast cancer patients [16], [17]. Ablation of *Mig-6* in mice leads to the development of epithelial hyperplasia, adenoma, and adenocarcinoma in organs, such as the uterus, lungs, gallbladder, and bile duct [17], [18], [19], [20], [21], [22], [23]. Attenuated *Mig-6* expression is thought to trigger cells to initiate hypertrophy in chronic pathological conditions, such as diabetes and hypertension [10], [24]. The expression of *Mig-6* was induced in hepatic cells by glucocorticoids and insulin [25], [26], [27]. Insulin-induced transcriptional increases in *Mig-6* are paralleled by increases in its protein product and are dependent upon insulin induction of the MEK-ERK signaling pathway [28]. Although *Mig-6* is expressed highest in the liver, to date, no hepatic phenotype has been reported in the *Mig-6^−/−^* mice. To investigate the metabolic role of *Mig-6* in the liver, we generated conditionally ablated *Mig-6* in the liver using the Albumin-Cre mouse model (*Alb^cre/+^Mig-6^f/f^*; *Mig-6^d/d^*). From this study, we found that *Mig-6* plays an important role in cholesterol homeostasis and bile acid synthesis.

## Materials and Methods

### Ethics statement

All animal research was conducted according to protocols approved by Institutional Animal Care and Use Committee (IACUC) of Baylor College of Medicine (approval ID number AN-4203).

### Animals and Tissue Collection

*Mig-6* “floxed” (*Mig-6^f/f^*) mice [19] and *Alb^cre/+^Mig-6^f/f^* (*Mig-6^d/d^*) mice were maintained in the designated animal care facility at the Baylor College of Medicine according to the institutional guidelines for the care and use of laboratory animals. Animals were maintained on a 12-h light/12-h dark cycle (0600 h/1800 h) and fed standard chow diet. Eight week old *Mig-6^f/f^* and *Mig-6^d/d^* male mice were assessed after undergoing a 24 hour fast. Mice were sacrificed by cervical dislocation after placing the mice under anesthesia, Avertin (2,2-tribromoethyl alcohol; Sigma-Aldrich, St. Louis, MO). The livers were weighed at the time of dissection, and the tissues were flash frozen and stored at −80°C.

### Serum and Tissue Chemistry

Serum was collected from the orbital sinus using disposable Pasteur pipets (Fisher Scientific, Pittsburgh, PA) after a 24 hour fasting period, placed in serum collecting tubes (BD, Franklin Lakes, NJ), centrifuged at 1,200× g for 10 min at 4°C, and stored at −20°C prior to analysis. Serum lipid profiles were analyzed by the Comparative Pathology Laboratory Center at Baylor College of Medicine. Feces were collected from individual mice for 3 days. Serum, liver, and fecal bile acid concentrations were measured by the ELISA method using the Bile Acids Assay kit (Diagnostic Chemicals Limited, Oxford, CT) according the manufacturers' instructions.

### Quantitative Real-Time RT-PCR

Total RNA was isolated using the Trizol reagent (Invitrogen, Carlsbad, CA). The expression level of genes was examined by real-time RT-PCR TaqMan analysis using the ABI Prism 7700 Sequence Detector System according to the manufacturer's instructions (PE Applied Biosystems, Foster City, CA). Prevalidated probes and primers for real-time PCR were purchased from Applied Biosystems (Table S1). mRNA quantities were normalized to 18S RNA using ABI rRNA control reagents. Statistical analyses used a two-tailed t test with the Instat package from GraphPad (San Diego, CA, USA). Probability values of *p*<0.05 were deemed significant.

### Microarray analysis

Microarray analysis was performed by the Affymetrix murine genome 430 ver 2.0 mouse oligonucleotide arrays (Affymetrix, Santa Clara, CA). All experiments were repeated 3 times. Briefly, we used DNA-Chip analyser dChip [29]. We selected differentially expressed genes within each time exposure using two sample comparisons according to the following criteria: lower bound of 90% confidence interval of fold change greater than 1.2 and an absolute value of difference between groups means greater than 50. After excluding expressed sequence tags with no functional annotation, differentially expressed genes were classified according to Gene Ontology function using Affymetrix annotation, literature search in PubMed, and Ingenuity Pathways Analysis (Ingenuity Systems Inc., Redwood City, CA). All data is MIAME compliant and the raw data has been deposited in a MIAME compliant database (NCBI's Gene Expression Omnibus). The microarray data is accessible through GEO series accession number GSE26913.

### Acylcarnitines and amino acids

For the analysis of acylcarnitines and amino acids, 8-week-old *Mig-6^f/f^* and *Mig-6^d/d^* mice (n = 5) were sacrificed after 24 hrs. of fasting, and proteins were first removed from the liver and serum by precipitation with methanol. Aliquoted supernatants were dried, and then esterified with hot acidic methanol (acylcarnitines) or n-butanol (amino acids). Acylcarnitines and amino acids were analyzed by tandem MS with a Quattro Micro instrument (Waters Corporation, Milford, MA). In total, 37 acylcarnitine species and 15 amino acids in plasma were assayed by previously described methods [30], [31], [32]. Leucine/isoleucine (LEU/ILE) are reported as a single analyte because they are not resolved by our MS/MS method, and include contributions from allo-isoleucine and hydroxyproline. Under normal circumstances, these isobaric amino acids contribute little to the signal attributed to LEU/ILE [33]. In addition, the acidic conditions used to form butyl esters results in partial hydrolysis of glutamine to glutamic acid and of asparagine to aspartate. Accordingly, values that are reported as GLU/GLN or ASP/ASN are not meant to signify the molar sum of glutamate and glutamine, or of aspartate and asparagine, but rather measure the amount of glutamate or aspartate plus the contribution of the partial hydrolysis reactions of glutamine and asparagine, respectively.

### Western Blot Analysis

Mouse liver tissues were washed with PBS solution and homogenized in a buffer containing 10 mM Tris-HCl (pH 7.4), 150 mM NaCl, 2.5 mM EDTA, and 0.125% Nonidet P-40 (vol/vol). Cellular debris was removed by centrifugation at 14,000 rpm for 15 min at 4°C. Protein concentration was determined by Bradford's method using BSA as the standard. Samples containing 30 µg proteins were applied to 10% SDS-PAGE. The separated proteins were then transferred onto a polyvinylidene difluoride membrane (Millipore Corp., Bedford, MA). Membranes were blocked overnight with 5% skim milk (wt/vol) in PBS with 0.1% Tween 20 (vol/vol) (Sigma-Aldrich, St. Louis, MO) and probed with rabbit-anti-Mig-6 (1∶1000, Sigma-Aldrich, St. Louis, MO). Immunoreactivity was visualized by incubation with a horse-radish peroxidase-linked second antibody and treatment with ECL reagents. To control for loading, the membrane was stripped, probed with mouse-anti-β-actin (1∶1000, Sigma-Aldrich, St. Louis, MO) and developed again.

### Tissue Staining

For Hematoxylin/Eosin (H&E) Staining, livers from 8 week old mice were fixed overnight in 4% paraformaldehyde, followed by thorough washing in 70% ethanol. The tissues were processed, embedded in paraffin, and sectioned. Five micrometer sections were cut and stained with hematoxylin and eosin by standard protocols.

For Oil Red O Staining, livers from 8 week old mice were fixed in 4% paraformaldehyde, frozen in OCT and sectioned. After 15 min air-dry, sections were stained with Oil-Red-O, counterstained with hematoxylin, and mounted with 15% glycerol.

## Results

### Generation of conditional ablation of *Mig-6* in the liver using *Alb^cre^*

**Figure 1:**
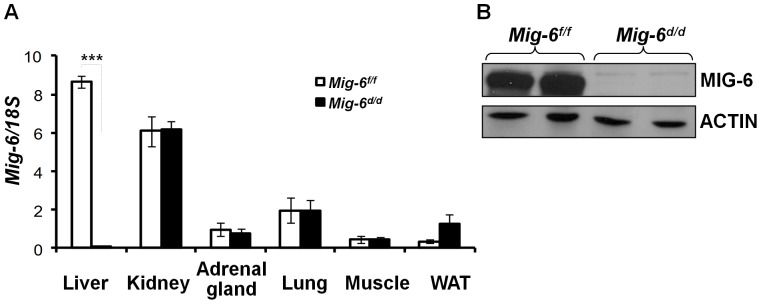
Generation of conditional ablation of *Mig-6* in the liver. A. RT-PCR analysis of *Mig-6* mRNA expression level. 8 week old *Mig-6^f/f^* and *Mig-6^d/d^* male mice were sacrificed after 24 hrs of fasting and RNA was isolated from the liver, kidney, adrenal gland, lungs, muscle, and white adipose tissue. Five mice of each group were used for this experiment. The results represent the mean ± SEM of three independent RNA sets. ***, *p*<0.001. B, Western blot analysis of MIG-6 in the liver of *Mig-6^f/f^* and *Mig-6^d/d^* mice. Liver tissue from *Mig-6^f/f^* and *Mig-6^d/d^* mice were lysed and equal amounts of protein were subjected to SDS-PAGE and Western blot analysis for MIG-6.

Since *Mig-6* ablation results in numerous pathologies and decreases in longevity [18], [21], [34], [35], our ability to investigate the role of *Mig-6* in the mouse liver is severely limited. Therefore, in order to investigate the role of *Mig-6* in the regulation of liver function and metabolism, *Mig-6^f/f^* mice [36] were bred to *Alb^Cre^* [37] mice to generate conditional *Mig-6* ablation (*Alb^cre^Mig-6^f/f^*; *Mig-6^d/d^*) in the liver. Liver-specific conditional *Mig-6* ablation was validated by examining the mRNA expression levels of *Mig-6* in the liver, kidney, adrenal gland, lungs, muscle, and white adipose tissue by real-time RT-PCR. The expression of *Mig-6* was strongly decreased in the liver of *Mig-6^d/d^* mice compared to *Mig-6^f/f^* mice. However, we could not observe any differences in the expression of the adrenal gland, kidney, lungs, muscle, and white adipose tissues of *Mig-6^d/d^* mice (Fig. 1A). Western blot analysis also confirmed a profound decrease in *Mig-6* protein in the liver of these mice (Fig. 1B). These results demonstrate that *Alb^cre^* efficiently decreased *Mig-6* expression in the liver.

### *Mig-6^d/d^* mice have hepatomegaly and increased intrahepatic lipid

**Figure 2:**
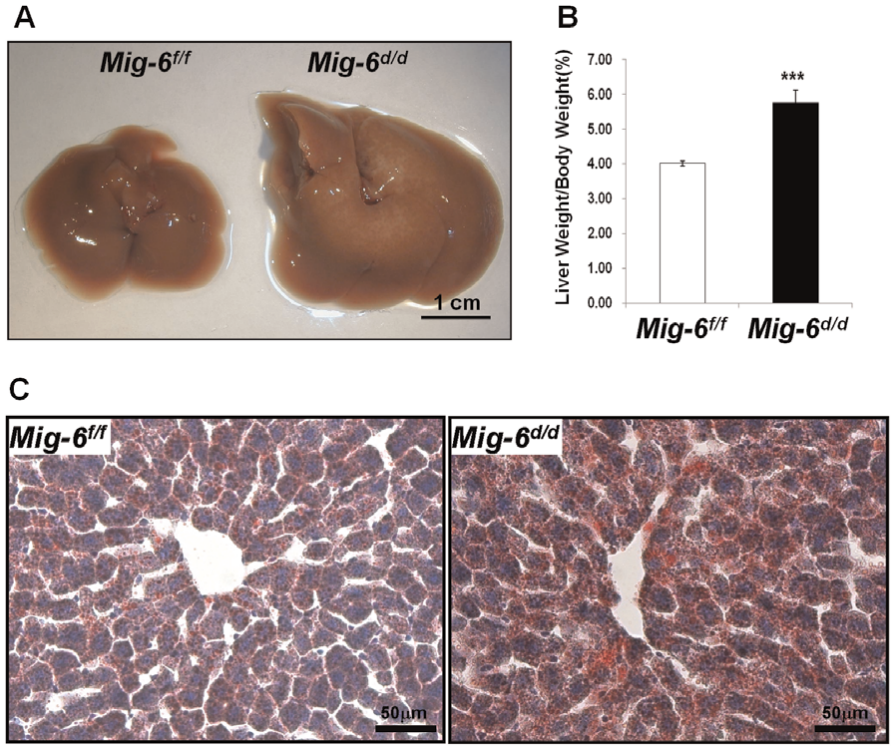
Morphology of the liver of *Mig-6^f/f^* and *Mig-6^d/d^* **mice.** A, Size of liver. Twelve mice of each group were used for this experiment. B, Weight of the liver adjusted to body weight. The results represent the mean ± SEM. The numbers in parentheses are the number of mice used. ***, p<0.001. C. Oil-Red-O staining. Liver tissue were fixed with 4% paraformaldehyde (vol/vol) and frozen in OCT. Sections were counterstained with hematoxylin and mounted with 15% glycerol. All of the photomicrographs are X400 magnification.

The size of the liver was enlarged in the *Mig-6^d/d^* mice compared to *Mig-6^f/f^* mice (Fig. 2A). The weight of the liver in 8-week-old *Mig-6^d/d^* mice was significantly increased compared to *Mig-6^f/f^* mice (Fig. 2B). However, there was not a difference in body weight between *Mig-6^f/f^* and *Mig-6^d/d^* mice. To further analyze the enlarged liver phenotype, the histology of the liver was examined. In H&E staining, many vacuolated lesions were found around the central vein of the *Mig-6^d/d^* liver (data not shown). Fat droplets were increased in the liver of *Mig-6^d/d^* compared to *Mig-6^f/f^* mice as shown by oil-red-O staining (Fig. 2C). These results demonstrate that *Mig-6^d/d^* mice have hepatomegaly and increased intrahepatic lipid.

### *Mig-6^d/d^* mice have hypercholesterolemia

To assess the function of *Mig-6* in the liver, 8-week-old *Mig-6^f/f^* and *Mig-6^d/d^* mice (n = 5) were sacrificed after 24 hrs. of fasting and the level of lipids was analyzed in the serum and liver (Table 1). *Mig-6^d/d^* mice displayed a 130.5% increase in serum total cholesterol level, which is a level that is significant when compared to *Mig-6^f/f^* mice. The levels of low density lipoprotein (LDL) cholesterol and high density lipoprotein (HDL) cholesterol were also significantly increased in the *Mig-6^d/d^* mice compared to *Mig-6^f/f^* mice (212.4% and 119.7%, respectively). However, serum triglyceride, very low density lipoprotein (VLDL), and bile acid levels were not changed between the *Mig-6^f/f^* and *Mig-6^d/d^* mice. The level of total cholesterol was 154.2% significantly increased in the liver of *Mig-6^d/d^* mice compared to *Mig-6^f/f^* mice (0.32±0.04 mg/g and 0.50±0.03 mg/g respectively). However, the level of triglyceride was not changed in serum and liver of *Mig-6^f/f^* and *Mig-6^d/d^* mice (0.95±0.05 mg/g and 1.25±0.11 mg/g respectively). We also determined the concentration of 60 serum metabolites, comprised of 15 amino acid and 45 acylcarnitines in *Mig-6^f/f^* and *Mig-6^d/d^* mice (n = 11). The specific analyses are summarized in Table S2. Of the acylcarnitines, the C5∶1, C10 (C10∶2 and C10∶3), C14 (C14∶1 and C14∶2), C16, C18 (C18, C18∶1, and C18∶2), C20∶4, C20∶1-OH/C18∶1-DC, and C22 are significantly increased in the serum of *Mig-6^d/d^* mice compared to *Mig-6^f/f^* mice. Among a total of 15 amino acids measures in the serum, arginine was significantly decreased in the *Mig-6^d/d^* mice. These serum chemistry results are in agreement with the observation that *Mig-6^d/d^* mice have hyperglycemia and hypercholesterolemia.

**Table 1:** Serum lipid profile in *Mig-6 ^f/f^* and *Mig-6^d/d^* mice.

	Total Cholesterol (mg/dl)	LDL Cholesterol (mg/dl)	HDL Cholesterol (mg/dl)	Triglyceride (mg/dl)	VLDL (mg/dl)
*Mig-6^f/f^*	143.0±21.8	12.9±3.0	135.0±21.1	59.8±18.1	11.9±3.6
*Mig-6^d/d^*	186.6±12.3**	27.4±5.6***	161.1±8.0*	69.0±9.7	13.8±1.9

Eight week old *Mig-6^d/d^* and *Mig-6^f/f^* male mice were sacrificed after 24 hrs of fasting and serum lipid profiles were analyzed. Five mice of each group were used for this experiment.

* , *p*<0.05;

** , *p*<0.01;

*** , *p*<0.001.

### *Mig-6* ablation altered the expression of genes related to lipid metabolism

In order to assess the impact of *Mig-6* ablation on liver mRNA expression profiles, we performed microarray analysis on *Mig-6^f/f^* and *Mig-6^d/d^* mice after 24 hrs. of fasting. Total RNA extracts were subjected to microarray analysis using the Affymetrix mouse genome 430 2.0 arrays. This analysis revealed 252 and 122 transcripts whose abundance was significantly increased or decreased, respectively, in the liver of *Mig-6^d/d^* mice as compared with *Mig-6^f/f^* controls. A complete list of the genes whose transcripts increase or decrease in abundance can be found in Tables S3 and S4, respectively. In order to determine which pathways are regulated by *Mig-6*, we performed pathway analysis using DAVID Analysis and Ingenuity Systems Software [38], [39]. The altered pathways included those involved in lipid metabolism, bile acid biosynthesis, biosynthesis of steroids, PPAR signaling pathway, citrate cycle (TCA cycle), and metabolism of xenobiotics by cytochrome P450. These pathways have all been implicated in metabolism suggesting that *Mig-6* is a critical mediator of lipid and bile acid metabolism in the liver.

**Figure 3:**
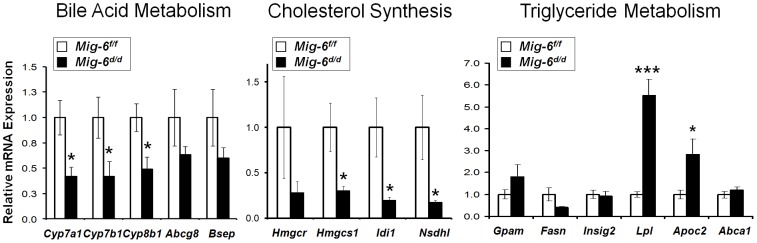
Real-time RT-PCR analysis of metabolic genes in the liver. A, Bile acid metabolism related genes. B, Cholesterol synthesis related genes. C, Triglyceride metabolism related genes. 8 week old *Mig-6^d/d^* and *Mig-6^f/f^* male mice were sacrificed after 24 hrs fasting and RNA was isolated from the liver. 5 mice of each group were used for this experiment. The results represent the mean ± SEM of three independent RNA sets. *, *p*<0.05; ***, *p*<0.001.

**Figure 4:**
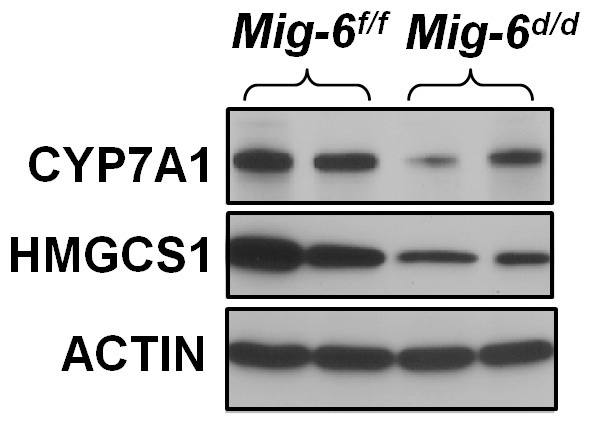
Regulation of CYP7A1 and HMGCS1 protein level in the liver of *Mig-6^d/d^* mice. Western blot analysis for CYP7A1 and HMGCS1 in the liver of *Mig-6^f/f^* and *Mig-6^d/d^* mice. Liver tissue from *Mig-6^f/f^* and *Mig-6^d/d^* mice were lysed, and equal amounts of protein were subjected to SDS-PAGE and Western blot analysis with anti-CYP7A1 and anti-HMGCS1 antibodies.

To further validate the microarray results and evaluate the hypercholesterolemia and fatty liver phenotypes, real-time RT-PCR was performed for several lipid and bile acid metabolism-related genes (Fig. 3). First, we examined genes involved in bile acid homeostasis. The mRNA expression of cholesterol 7-α-hydroxylase (*Cyp7a1*), the rate-limiting enzyme for classic pathway of bile acid synthesis, as well as oxysterol 7-α-hydroxylase 1 (*Cyp7b1*) and sterol 12-α-hydroxylase (*Cyp8b1*), which are also involved in bile acid synthesis, were decreased in *Mig-6^d/d^* mice. While genes in biliary excretion of bile acid were not identified in the microarray, we assessed the expression of two genes shown to be involved in this process, ATP-binding cassette, Subfamily G, Member 8 (*Abcg8*) and Bile salt export pump (*Bsep*), and found that their expression was not different between the two groups. This indicates that *Mig-6* is involved in the regulation of specific enzymes involved in bile acid synthesis, but does not regulate their excretion. With respect to cholesterol synthesis, the expression of certain synthetic enzymes (3-hydroxy-3-methylglutaryl-Coenzyme A synthethase 1 (*Hmgcs1*), isopentenyl-diphosphate-isomerase (*Idi1*) and NAD(P)H steroid dehydrogenase-like protein (*Nsdhl*)) were significantly decreased in the *Mig-6^d/d^* mice compared to *Mig-6^f/f^* mice while others (3-hydroxy-3-methylglutaryl-Coenzyme A reductase (*Hmgcr*)) were not. The expression of genes involved in cholesterol and bile acid metabolism such as liver X receptor (*Lxr*) alpha and beta, liver receptor homolog-1 (*Lrh-1*), inducible degrader of the low density lipoprotein receptor (*Idol*), small heterodimer partner (*Shp*), and farnesoid X-activated receptor (*Fxr*) were not altered in the *Mig-6^d/d^* mice. The mRNA expression of lipoprotein lipase (*Lpl*) and apolipoprotein-CII (*ApoC2*), which are involved in lipolysis, were increased in the *Mig-6^d/d^* mice. The expression of other genes involved in triglyceride metabolism (glycerol-3-phosphate acyltransferase, mitochondrial (*Gpam*) , fatty acid synthase (*Fasn*), insulin induced gene 2 (*Insig2*), and ATP-binding cassette, sub-family A (*ABC1*), member 1 (*Abca1*)) were not altered in the *Mig-6^d/d^* mice. We also confirmed the protein expression profile for lipid metabolism related proteins. We investigated the expression level of CYP7A1 and HMGCS1 proteins in the mouse liver by employing western blot analysis. As shown by Western blot analyses using specific CYP7A1 and HMGCS1 antibodies, levels of CYP7A1 and HMGCS1 in the *Mig-6^d/d^* mice were lower than *Mig-6^f/f^* control mice (Fig. 4). These results are consistent with our phenotypic analysis indicating that *Mig-6^d/d^* mice have a defect in lipid and bile acid metabolism in their liver.

### Fecal excretion of bile acid is significantly decreased in *Mig-6^f/f^* mice

Hypercholesterolemia is the presence of high levels of cholesterol in the blood [40]. It is a metabolic derangement that can be secondary to many diseases and can contribute to many diseases, most notably cardiovascular disease. To address whether bile acid metabolism is responsible for the hypercholesterolemia phenotype, the concentration of bile acids was measured in the serum, liver tissue, and feces from *Mig-6^f/f^* and *Mig-6^d/d^* mice. The concentration of bile acids in the serum and liver tissue was not significantly different between the two groups. Daily excretion of fecal bile acids in the *Mig-6^d/d^* mice was about 33.8% (p<0.01) lower than the *Mig-6^f/f^* mice (Table 2). These results indicate that the fecal excretion of bile acids was altered in the *Mig-6^d/d^* mice.

**Table 2:** Concentration of bile acid in serum, liver, and feces.

	Bile Acid		
	Serum(µmol/L)	Liver(µmol/mg tissue)	Feces(µmol/day/kg body weight)
*Mig-6^f/f^*	11.6±0.62	233.7±16.5	105.4±3.1
*Mig-6^d/d^*	10.7±0.48	222.7±26.5	69.8±7.5**

Serum and liver were collected from 8 week old *Mig-6^d/d^* and *Mig-6^f/f^* male mice after 24 hrs. of fasting. Feces were collected from individual mice for 3 days. 5 mice of each group were used for this experiment.

** , *p*<0.01.

## Discussion

Since *Mig-6* ablation results in a 50% reduction of the *Mig-6^−/−^* litter size and in numerous pathologies and decreased longevity [18], [21], [34], [35], our ability to investigate the role of *Mig-6* in the liver is severely limited. In order to effectively investigate the role of *Mig-6* in the regulation of liver function, we generated conditional *Mig-6* ablation in the liver using the *Alb^cre^* mouse model. We validated that this ablation occurred specifically in the liver and then assessed the liver phenotype (Fig. 1).

One of the major functions of the liver is the control of nutrient metabolism including cholesterol [41], [42]. We found that *Mig-6^d/d^* mice have abnormalities related to cholesterol metabolism, such as hypercholesterolemia, characterized by markedly increased LDL cholesterol, increased intrahepatic lipid, and hepatomegaly. The four major causes of hypercholesterolemia can be classified as an increased intake of cholesterol in the diet, increased synthesis of cholesterol in the liver, decreased excretion of cholesterol as bile acid, and decreased uptake of cholesterol from the blood to the liver [5], [43], [44]. The expressions of genes that were related to cholesterol synthesis, such as *Idi1* and *Nsdhl*, were significantly decreased in the *Mig-6^d/d^* mice. While these changes in gene expression are different from what would be expected by examining the liver of these mice, they may be explained as potential compensatory mechanisms to counteract the hypercholesterolemia. Cholesterol synthesis can be decreased when cholesterol levels are high. HMG CoA reductase contains both a cytosolic domain and a membrane domain. The membrane domain functions to sense signals for its degradation. Increasing concentrations of cholesterol cause a change in this domain's oligomerization state, which makes it more susceptible to destruction by the proteosome.

The concentration of bile acid in the serum and liver were unchanged, however, bile acid in the feces was decreased in the *Mig-6^d/d^* mice indicating that fecal excretion of cholesterol as bile acid was altered. However, the expression level of genes related to biliary excretion of bile acids, such as *Abcg8* and *Bsep*, were unchanged indicating that there may be alterations in the bile acid pool. The results of metabolomics analysis suggest that serum level of acylcarnitines was increased in the *Mig-6^d/d^* mice compared to *Mig-6^f/f^* mice (Table S2). An increase of acrylarnitines explains the hepatic steatosis phenotype in the *Mig-6^d/d^* mice because acylcarnitine level reflect fatty acid beta oxidation [45]. Overall, these data suggest that the cause of hypercholesterolemia is related to decrease fecal excretion of cholesterol as bile acid.

Bile acid is critical for the maintenance of cholesterol homeostasis and is essential for the absorption of dietary lipids, lipid soluble vitamins, and other nutrients [43], [44], [46]. Maintenance of the enterohepatic bile acid pool is important for these functions [47]. Defects in bile acid synthesis cause several human diseases, such as cerebrotendinous xanthomatosis [44]. The enterohepatic bile acid pool is maintained by the balance between the synthesis of bile acids in the liver and by the excretion of bile acids in the feces. When the synthesis of bile acids decreases, the body decreases fecal bile acid excretion as compensation in order to maintain the bile acid pool [46].

*Cyp7a1* and *Cyp8b1* are the major regulatory enzymes for the classic bile acid synthesis pathway and *Cyp7b1* is used in the alternative pathway of bile acid synthesis [43], [44], [46]. The mRNA expression of these genes decreased in the *Mig-6^d/d^* mice. Thus, bile acid synthesis is decreased in the liver. However, normal levels of liver bile acids were observed. This is likely due to the compensation of decreased fecal excretion of bile acids by the liver. *Cyp7a1* was one gene that was deregulated in the liver upon ablation of *Mig-6*. The formation of bile acids is of major importance for the maintenance of cholesterol homeostasis. *Cyp7a1* is expressed only in the liver and is a rate-limiting enzyme in bile acid synthesis and cholesterol catabolism [43], [44], [48]. *Cyp7a1* has been implicated in genetic susceptibility to atherosclerosis. A null mutation in human *CYP7A1* is characterized by a significantly increased total and LDL cholesterol levels, substantial accumulation of cholesterol in the liver and a markedly decreased rate of bile acid excretion [49]. *Cyp7a1*-deficient mice display hypercholesterolemia, fatty liver, and hepatomegaly [48]. They also exhibit decreased fecal excretion of bile acids. As these phenotypes are similar to that of the *Mig-6^d/d^* mice, *Cyp7a1* is likely a key mediator of the phenotypes observed upon ablation of *Mig-6* in the liver. The expression of *Cyp7a1* is inhibited by a mechanism involving the bile acid-activated farnesoid X receptor (FXR) in hepatocytes and enterocytes. Activation of FXR induced small heterodimer partner (SHP) to inhibit *Cyp7a1* gene transcription in the liver [50]. However, the expression of LXR, LRH1, SHP, and FXR were not altered in this study. Because *Mig-6* is an adaptor protein, we predict that this regulation occurs via protein-protein interactions with an unidentified signaling pathway and/or transcription factor. The mechanism behind *Mig-6*'s regulation of *Cyp7a1* remains unknown.

*Mig-6* is a negative regulator for growth signals such as epidermal, hepatocyte, and platelet-drived growth factors. The expression of *Mig-6* is induced by insulin [25], [26], [27]. Insulin is a major postprandial factor that induces *Cyp7a1* gene expression and bile acid synthesis. Bile acids are the end products of cholesterol catabolism in the liver [51]. In addition to the classic function of bile acids in facilitating intestine absorption and transport of nutrients, drugs, and steroids, bile acids also play important roles in regulating the lipids, drugs, glucose, and energy metabolism [6]. Bile acid synthesis is mainly controlled by the transcriptional regulation of the *Cyp7a1* gene. The overexpression of *Cyp7a1* have increased bile acid pool and are resistant to high fat diet-induced insulin resistance and obesity [52]. This result suggests that bile acid signaling is important in maintaining metabolic homeostasis. Impaired bile acid homeostasis could lead to adverse metabolic consequences, such as cholestasis, liver injury, diabetes, and obesity [52]. Bile acids facilitate postprandial absorption of nutrients and play a major role in regulating lipid, glucose, and energy metabolism. These suggest that dysregulations of *Cyp7a1* expression and bile acid metabolism may play a critical for the phenotypes in the *Mig-6^d/d^* mice.

In summary, *Mig-6* ablation in the liver results in multiple metabolic phenotypes. The *Mig-6^d/d^* mice exhibit hepatomegdaly and increased intrahepatic lipid. There is an increase in level of serum cholesterol. This hypercholesterolemia is likely due to decreased bile acid synthesis which resulted in decreased fecal excretion of bile acids and an overall disruption of cholesterol homeostasis. Hypercholesterolemia is one of the major modifiable causes of cardiovascular disease [1], [2], [3]. Defining the molecular mechanisms by which *Mig-6* regulates cholesterol homeostasis will provide new insight into the development of more effective ways for the treatment and prevention of hypercholesterolemia and, therefore, cardiovascular disease.

## Supporting Information

Table S1 **Applied biosystems assay identification for real time RT-PCR analysis.**
(PDF)

Table S2 **The concentration of metabolites in serum of** ***Mig-6^f/f^*** **and** ***Mig-6^d/d^*** **mice.** *, *p*<0.05, **, *p*<0.01.
(PDF)

Table S3 **The significantly decreased genes in the liver of** ***Mig-6^d/d^*** **mice as compared to** ***Mig-6^f/f^*** **mice.**
(PDF)

Table S4 **The significantly increased genes in the liver of** ***Mig-6^d/d^*** **mice as compared to** ***Mig-6^f/f^*** **mice.**
(PDF)
