# Supplementary material for: Mig-6 Plays a Critical Role in the Regulation of Cholesterol Homeostasis and Bile Acid Synthesis
Source: PLoS One. 2012 Aug 17;7(8):e42915. doi: 10.1371/journal.pone.0042915 (PMC3422237; doi:10.1371/journal.pone.0042915)
Supplement: Table S1 — Applied biosystems assay identification for real time RT-PCR analysis. (PDF) [file pone.0042915.s001.pdf]

Table S1. Applied biosystems assay identification for real-time RT-PCR analysis

**Gene**	**Assay Identification**
Mig-6	Mm00505292_m1
Cyp7a1	Mm00484152_m1
Cyp7b1	Mm00484157_m1
Cyp8b1	Mm00501637_s1
Abcg8	Mm00445970_m1
Bsep	Mm00445168_m1
Hmgcr	Mm01282499_m1
Idi1	Mm01337454_m1
Nsdhl	Mm00477897_m1
Ppat	Mm00549096_m1
Fasn	Mm00662319_m1
Insig2	Mm00460121_m1
Lpl	Mm00434764_m1
Apoc2	Mm00437571_m1
Abca1	Mm00442646_m1
18S ribosomal RNA	Mm03928990_g1
