# Supplementary material for: Mig-6 Plays a Critical Role in the Regulation of Cholesterol Homeostasis and Bile Acid Synthesis
Source: PLoS One. 2012 Aug 17;7(8):e42915. doi: 10.1371/journal.pone.0042915 (PMC3422237; doi:10.1371/journal.pone.0042915)
Supplement: Table S2 — The concentration of metabolites in serum of Mig-6f/f and Mig-6d/d mice. *, p<0.05, **, p<0.01. (PDF) [file pone.0042915.s002.pdf]

Table S2. The concentration of metabolites in serum of *Mig-6*$^{f/f}$ and *Mig-6*$^{d/d}$ mice.

**Amino Acid** (μM)	*Mig-6*$^{f/f}$			*Mig-6*$^{d/d}$			Fold change	p-value
Gly	312.74	±	14.15	322.96	±	12.81	0.97	0.5983
Ala	768.92	±	31.25	743.01	±	48.94	1.03	0.6603
Ser	198.71	±	10.21	197.81	±	8.31	1.00	0.9462
Pro	131.09	±	5.55	140.91	±	8.77	0.93	0.3560
Val	256.32	±	12.84	245.54	±	11.90	1.04	0.5447
Leu/Ile	266.90	±	16.07	257.46	±	14.63	1.04	0.6686
Met	80.64	±	4.25	90.70	±	4.13	0.89	0.1054
His	81.13	±	4.62	79.35	±	2.94	1.02	0.7475
Phe	103.48	±	4.83	104.57	±	5.21	0.99	0.8792
Tyr	94.08	±	4.21	93.22	±	4.01	1.01	0.8832
Asx	21.34	±	2.99	26.75	±	3.05	0.80	0.2196
Glx	163.79	±	12.40	182.63	±	9.75	0.90	0.2464
Orn	212.99	±	39.86	165.39	±	15.67	1.29	0.2795
Cit	70.89	±	3.57	74.38	±	2.91	0.95	0.4576
Arg	193.01	±	21.96	275.38	±	28.64	0.70*	0.0336

**Acylcarnitines** (μM)	*Mig-6*$^{f/f}$			*Mig-6*$^{d/d}$			Fold change	p-value
C2	22.7152	±	2.4630	23.1025	±	1.8344	0.9832	0.9009
C3	0.4898	±	0.0626	0.4674	±	0.0442	1.0479	0.7730
C4/Ci4	0.4688	±	0.0575	0.4063	±	0.0305	1.1539	0.3485
C5:1	0.1147	±	0.0076	0.0917	±	0.0033	1.2504*	0.0113
C5's	0.3582	±	0.0701	0.3281	±	0.0343	1.0916	0.7044
C4-OH	0.2026	±	0.0248	0.2206	±	0.0140	0.9186	0.5352
C6	0.0629	±	0.0250	0.0348	±	0.0158	1.8087	0.3523
C5-OH/C3-DC	0.2079	±	0.0117	0.2483	±	0.0151	0.8373*	0.0473
Ci4-DC/C4-DC	0.0488	±	0.0037	0.0555	±	0.0046	0.8794	0.2669
C8:1	0.0136	±	0.0016	0.0141	±	0.0017	0.9658	0.8374
C8	0.0175	±	0.0012	0.0158	±	0.0015	1.1077	0.3930
C5-DC	0.0218	±	0.0024	0.0189	±	0.0012	1.1511	0.3043
C6:1-DC/C8:1-OH	0.0049	±	0.0006	0.0039	±	0.0003	1.2653	0.1176
C6-DC	0.0224	±	0.0029	0.0222	±	0.0023	1.0120	0.9432
C10:3	0.0351	±	0.0033	0.0271	±	0.0019	1.2940*	0.0484
C10:2	0.0058	±	0.0005	0.0039	±	0.0004	1.4876**	0.0046
C10:1	0.0211	±	0.0014	0.0195	±	0.0021	1.0837	0.5188
C10	0.0326	±	0.0084	0.0156	±	0.0082	2.0893	0.1612
C7-DC	0.0394	±	0.0100	0.0272	±	0.0111	1.4480	0.4227
C8:1-DC	0.0061	±	0.0007	0.0058	±	0.0007	1.0489	0.7832

**Acylcarnitines** (μM)	*Mig-6*$^{f/f}$			*Mig-6*$^{d/d}$			Fold change	p-value
C10-OH/C8-DC	0.0136	±	0.0016	0.0157	±	0.0012	0.8614	0.2840
C12:1	0.0200	±	0.0015	0.0173	±	0.0021	1.1609	0.2937
C12	0.0338	±	0.0024	0.0329	±	0.0034	1.0273	0.8300
C12-OH/C10-DC	0.0033	±	0.0004	0.0034	±	0.0005	0.9670	0.8669
C14:2	0.0323	±	0.0016	0.0247	±	0.0028	1.3071*	0.0311
C14:1	0.0813	±	0.0054	0.0593	±	0.0065	1.3713*	0.0163
C14	0.0757	±	0.0055	0.0599	±	0.0055	1.2639	0.0557
C14:1-OH/C12:1-DC	0.0192	±	0.0016	0.0160	±	0.0019	1.1988	0.2114
C14-OH/C12-DC	0.0075	±	0.0009	0.0066	±	0.0014	1.1364	0.5924
C16:2	0.0204	±	0.0017	0.0169	±	0.0021	1.2054	0.2095
C16:1	0.0556	±	0.0045	0.0430	±	0.0050	1.2913	0.0783
C16	0.2562	±	0.0185	0.1967	±	0.0143	1.3022*	0.0193
C16:1-OH/C14:1-DC	0.0116	±	0.0008	0.0105	±	0.0008	1.1063	0.3474
C16-OH/C14-DC	0.0098	±	0.0009	0.0085	±	0.0009	1.1483	0.3299
C18:2	0.1551	±	0.0111	0.1152	±	0.0127	1.3468*	0.0280
C18:1	0.2486	±	0.0198	0.1680	±	0.0169	1.4799**	0.0056
C18	0.0882	±	0.0052	0.0645	±	0.0046	1.3683**	0.0028
C18:2-OH	0.0109	±	0.0013	0.0081	±	0.0016	1.3425	0.1925
C18:1-OH/C16:1-DC	0.0163	±	0.0020	0.0134	±	0.0015	1.2157	0.2675
C18-OH/C16-DC	0.0172	±	0.0019	0.0132	±	0.0015	1.3030	0.1184
C20:4	0.0352	±	0.0031	0.0259	±	0.0024	1.3625*	0.0286
C20	0.0087	±	0.0010	0.0063	±	0.0010	1.3785	0.0958
C20:1-OH/C18:1-DC	0.0062	±	0.0002	0.0042	±	0.0007	1.4735*	0.0158
C20-OH/C18-DC	0.0019	±	0.0005	0.0011	±	0.0003	1.7013	0.1886
C22	0.0047	±	0.0006	0.0024	±	0.0007	1.9329*	0.0273

*, $p < 0.05$, **, $p < 0.01$
