# Supplementary material for: Mig-6 Plays a Critical Role in the Regulation of Cholesterol Homeostasis and Bile Acid Synthesis
Source: PLoS One. 2012 Aug 17;7(8):e42915. doi: 10.1371/journal.pone.0042915 (PMC3422237; doi:10.1371/journal.pone.0042915)
Supplement: Table S3 — The significantly decreased genes in the liver of Mig-6d/d mice as compared to Mig-6f/f mice. (PDF) [file pone.0042915.s003.pdf]

Table S3. The significantly decreased genes in the liver of *Mig-6*$^{d/d}$ mice as compared to *Mig-6*$^{f/f}$ mice

**Probe set**	**Gene**	**Gene Title**	**Fold change**
1416129_at	Mig-6	Mitogen inducible gene 6	-86.83
1419816_s_at	Mig-6	Mitogen inducible gene 6	-48.83
1418918_at	Igfbp1	insulin-like growth factor binding protein 1	-4.54
1428223_at	Mfsd2	major facilitator superfamily domain containing 2	-3.78
1422643_at	Moxd1	monooxygenase, DBH-like 1	-3.46
1448700_at	G0s2	G0/G1 switch gene 2	-3.30
1423418_at	Fdps	farnesyl diphosphate synthetase	-3.27
1432517_a_at	Nnmt	nicotinamide N-methyltransferase	-3.03
1448510_at	Efna1	ephrin A1	-2.98
1424735_at	Slc25a25	solute carrier family 25 (mitochondrial carrier, phosphate carrier), member 25	-2.97
1437867_at		Transcribed locus, strongly similar to XP_001478479.1	-2.68
1423078_a_at	Sc4mol	sterol-C4-methyl oxidase-like	-2.67
1451122_at	Idi1	isopentenyl-diphosphate delta isomerase	-2.65
1450611_at	Orm3	orosomucoid 3	-2.57
1416895_at	Efna1	ephrin A1	-2.55
1453023_at	Ankhd1	eukaryotic translation initiation factor 4E binding protein 3	-2.51
1436966_at	Peli2	pellino 2	-2.42
1417880_at	G6pc	glucose-6-phosphatase, catalytic	-2.40
1448975_s_at	Ren1	renin 1 structural	-2.39
1419874_x_at	Zbtb16	zinc finger and BTB domain containing 16	-2.39
1421040_a_at	Gsta2	glutathione S-transferase, alpha 2 (Yc2)	-2.37
1441430_at		Transcribed locus	-2.37
1427981_a_at	Csad	cysteine sulfinic acid decarboxylase	-2.30
1442025_a_at		Transcribed locus, strongly similar to NP_001028496.1	-2.27
1452975_at	Agxt2l1	alanine-glyoxylate aminotransferase 2-like 1	-2.24
1448986_x_at		deoxyribonuclease II alpha	-2.19
1416630_at	Id3	inhibitor of DNA binding 3	-2.13
1450970_at	Got1	glutamate oxaloacetate transaminase 1, soluble	-2.11
1449309_at	Cyp8b1	cytochrome P450, family 8, subfamily b, polypeptide 1	-2.09
1433443_a_at	Hmgcs1	3-hydroxy-3-methylglutaryl-Coenzyme A synthase 1	-2.08
1422100_at	Cyp7a1	cytochrome P450, family 7, subfamily a, polypeptide 1	-2.08
1427797_s_at		Mouse endogenous murine mink cell focus-forming (MCF) envelope protein mRNA	-2.07
1438743_at	Cyp7a1	cytochrome P450, family 7, subfamily a, polypeptide 1	-2.06
1417409_at	Jun	Jun oncogene	-2.06
1421041_s_at	Gsta1	glutathione S-transferase, alpha 1 (Ya)	-2.05
1424937_at		RIKEN cDNA 2310076L09 gene	-2.05
1435779_at	Cep110	centrosomal protein 110	-2.04
1457374_at	Nedd4l	neural precursor cell expressed, developmentally down-regulated gene 4-like	-2.03
1429144_at	Prei4	preimplantation protein 4	-2.03
1416411_at	Gstm2	glutathione S-transferase, mu 2	-2.00
1456464_x_at	Syt11	Synaptotagmin XI	-2.00
1422474_at	Pde4b	phosphodiesterase 4B, cAMP specific	-1.98
1424405_at	Mbip	MAP3K12 binding inhibitory protein 1	-1.96

Probe set	Gene	Gene Title	Fold change
1439163_at	Zbtb16	zinc finger and BTB domain containing 16	-1.96
1416222_at	Nsdhl	NAD(P) dependent steroid dehydrogenase-like	-1.92
1416933_at	Por	P450 (cytochrome) oxidoreductase	-1.90
1420541_at	Rdh16	retinol dehydrogenase 16	-1.89
1457248_x_at	Hsd17b7	hydroxysteroid (17-beta) dehydrogenase 7	-1.88
1455025_at	Paqr9	progestin and adipoQ receptor family member IX	-1.88
1421074_at	Cyp7b1	cytochrome P450, family 7, subfamily b, polypeptide 1	-1.84
1417981_at	Insig2	insulin induced gene 2	-1.84
1450717_at	Ang	angiogenin, ribonuclease, RNase A family, 5	-1.83
1420615_at	Ash2l	ash2 (absent, small, or homeotic)-like (Drosophila)	-1.83
1421075_s_at	Cyp7b1	cytochrome P450, family 7, subfamily b, polypeptide 1	-1.82
1416452_at	Oat	ornithine aminotransferase	-1.82
1446463_at	Adnp2	ADNP homeobox 2	-1.80
1418932_at		nuclear factor, interleukin 3, regulated	-1.80
1441915_s_at		RIKEN cDNA 2310076L09 gene	-1.80
1421622_a_at	Rapgef4	Rap guanine nucleotide exchange factor (GEF) 4	-1.77
1456295_at		RIKEN cDNA B230114P17 gene	-1.77
1419144_at	Cd163	CD163 antigen	-1.75
1425518_at	Rapgef4	Rap guanine nucleotide exchange factor (GEF) 4	-1.74
1452889_at		RIKEN cDNA 2310007H09 gene	-1.73
1417982_at	Insig2	insulin induced gene 2	-1.71
1425921_a_at		RIKEN cDNA 1810055G02 gene	-1.68
1436168_at		RIKEN cDNA C730029A08 gene	-1.67
1426215_at	Ddc	dopa decarboxylase	-1.65
1417980_a_at	Insig2	insulin induced gene 2	-1.65
1438937_x_at	Ang	angiogenin, ribonuclease, RNase A family, 5	-1.64
1448865_at	Hsd17b7	hydroxysteroid (17-beta) dehydrogenase 7	-1.64
1422603_at	Rnase4	ribonuclease, RNase A family 4	-1.64
1425077_at	Dnajc18	DnaJ (Hsp40) homolog, subfamily C, member 18	-1.63
1435031_at	Tmem120a	transmembrane protein 120A	-1.63
1420836_at	Slc25a30	solute carrier family 25, member 30	-1.61
1418603_at	Avpr1a	arginine vasopressin receptor 1A	-1.60
1423523_at	Aass	aminoadipate-semialdehyde synthase	-1.59
1448318_at	Adfp	adipose differentiation related protein	-1.57
1455227_at	Aadacl1	arylacetamide deacetylase-like 1	-1.57
1426648_at	Mapkapk2	MAP kinase-activated protein kinase 2	-1.57
1456767_at	Lrfn3	leucine rich repeat and fibronectin type III domain containing 3	-1.55
1453043_at		RIKEN cDNA 0610012H03 gene	-1.55
1433898_at		Transcribed locus	-1.55
1423447_at	Clpx	caseinolytic peptidase X (E.coli)	-1.54
1424828_a_at	Fh1	fumarate hydratase 1	-1.54
1449125_at	Tnfaip8l1	tumor necrosis factor, alpha-induced protein 8-like 1	-1.54
1436169_at		Transcribed locus	-1.53
1450644_at	Zfp36l1	zinc finger protein 36, C3H type-like 1	-1.53
1420499_at	Gch1	GTP cyclohydrolase 1	-1.52

Probe set	Gene	Gene Title	Fold change
1452653_at	Slc25a22	solute carrier family 25 (mitochondrial carrier, glutamate), member 22	-1.52
1451286_s_at	Fus	fusion, derived from t(12;16) malignant liposarcoma (human)	-1.51
1456194_a_at	Park7	Parkinson disease (autosomal recessive, early onset) 7	-1.51
1460684_at	Tm7sf2	transmembrane 7 superfamily member 2	-1.51
1427347_s_at	Tubb2a	tubulin, beta 2a	-1.51
1449047_at	Hacl1	2-hydroxyacyl-CoA lyase 1	-1.50
1423130_a_at	Sfrs5	splicing factor, arginine/serine-rich 5 (SRp40, HRS)	-1.50
1448350_at	Asl	argininosuccinate lyase	-1.49
1452832_s_at	Cds2	CDP-diacylglycerol synthase (phosphatidate cytidylyltransferase) 2	-1.48
1452730_at	Rps4y2	ribosomal protein S4, Y-linked 2	-1.48
1420966_at	Slc25a15	solute carrier family 25 (mitochondrial carrier ornithine transporter), member 15	-1.48
1450395_at	Slc22a5	solute carrier family 22 (organic cation transporter), member 5	-1.45
1449492_a_at	Lect2	leukocyte cell-derived chemotaxin 2	-1.44
1434592_at	Slc16a10	solute carrier family 16 (monocarboxylic acid transporters), member 10	-1.43
1416530_a_at		purine-nucleoside phosphorylase 1	-1.41
1417232_at	Cldn2	claudin 2	-1.40
1460673_at	Fpgs	folylpolyglutamyl synthetase	-1.40
1416593_at	Glrx	glutaredoxin	-1.40
1430570_at	Kynu	kynureninase (L-kynurenine hydrolase)	-1.40
1416526_a_at	Park7	Parkinson disease (autosomal recessive, early onset) 7	-1.40
1431197_at	Arl6ip2	ADP-ribosylation factor-like 6 interacting protein 2	-1.39
1416772_at	Cpt2	carnitine palmitoyltransferase 2	-1.39
1460241_a_at	St3gal5	ST3 beta-galactoside alpha-2,3-sialyltransferase 5	-1.38
1415948_at	Creg1	cellular repressor of E1A-stimulated genes 1	-1.37
1448330_at	Gstm1	glutathione S-transferase, mu 1	-1.37
1439617_s_at	Pck1	phosphoenolpyruvate carboxykinase 1, cytosolic	-1.37
1422780_at	Pxmp4	peroxisomal membrane protein 4	-1.36
1432016_a_at	Idh3a	isocitrate dehydrogenase 3 (NAD+) alpha	-1.35
1416794_at	Arl6ip2	ADP-ribosylation factor-like 6 interacting protein 2	-1.34
1417590_at	Cyp27a1	cytochrome P450, family 27, subfamily a, polypeptide 1	-1.33
1451297_at	Gulo	gulonolactone (L-) oxidase	-1.33
1448348_at	Caprin1	cell cycle associated protein 1	-1.32
1428821_at	Agpat2	1-acylglycerol-3-phosphate O-acyltransferase 2 (lysophosphatidic acid acyltransferase, beta)	-1.30
1450957_a_at	Sqstm1	sequestosome 1	-1.30
