# Supplementary material for: Mig-6 Plays a Critical Role in the Regulation of Cholesterol Homeostasis and Bile Acid Synthesis
Source: PLoS One. 2012 Aug 17;7(8):e42915. doi: 10.1371/journal.pone.0042915 (PMC3422237; doi:10.1371/journal.pone.0042915)
Supplement: Table S4 — The significantly increased genes in the liver of Mig-6d/d mice as compared to Mig-6f/f mice. (PDF) [file pone.0042915.s004.pdf]

Table S4. The significantly increased genes in the liver of *Mig-6*$^{d/d}$ mice as compared to *Mig-6*$^{f/f}$ mice

Probe set	Gene	Gene Title	Fold change
1417370_at	Tff3	trefoil factor 3, intestinal	7.88
1441799_at		RIKEN cDNA 6030422H21 gene	6.05
1419590_at	Cyp2b9	cytochrome P450, family 2, subfamily b, polypeptide 9	4.97
1456960_at		Mus musculus 2 days pregnant adult female ovary cDNA	4.49
1444438_at	Cib3	calcium and integrin binding family member 3	4.42
1458089_at		Transcribed locus	4.34
1415904_at	Lpl	lipoprotein lipase	4.27
1438558_x_at	Foxq1	forkhead box Q1	4.21
1431916_at	Hsd3b3	hydroxy-delta-5-steroid dehydrogenase, 3 beta- and steroid delta-isomerase 3	3.85
1426037_a_at	Rgs16	regulator of G-protein signaling 16	3.79
1431056_a_at	Lpl	lipoprotein lipase	3.63
1440847_at	Mtss1	metastasis suppressor 1	3.47
1446284_at	Mtss1	metastasis suppressor 1	3.45
1438431_at	Abcd2	ATP-binding cassette, sub-family D (ALD), member 2	3.41
1455265_a_at	Rgs16	regulator of G-protein signaling 16	3.40
1447040_at		Transcribed locus	3.33
1419748_at	Abcd2	ATP-binding cassette, sub-family D (ALD), member 2	3.21
1443949_at		RIKEN full-length enriched library, clone:9530006N05	2.98
1429159_at	Itih5	inter-alpha (globulin) inhibitor H5	2.79
1459948_at		Mus musculus, clone IMAGE:1511633, mRNA	2.75
1422735_at	Foxq1	forkhead box Q1	2.71
1448754_at		retinol binding protein 1, cellular	2.71
1438211_s_at	Dbp	D site albumin promoter binding protein	2.70
1453939_x_at		similar to ISG15 ubiquitin-like modifier	2.69
1443086_at	Alcam	activated leukocyte cell adhesion molecule	2.68
1459238_at		Mus musculus transcribed sequences	2.59
1425303_at	Gck	glucokinase	2.53
1458099_at		RIKEN full-length enriched library, clone:B130004P22	2.52
1453588_at	Car3	carbonic anhydrase 3	2.51
1456973_at		RIKEN full-length enriched library, clone:9930108O06	2.51
1421092_at	Serpina12	serine (or cysteine) peptidase inhibitor, clade A (alpha-1 antiproteinase, antitrypsin), member 12	2.48
1443056_at		RIKEN full-length enriched library, clone:C130078A05	2.48
1449254_at	Spp1	secreted phosphoprotein 1	2.47
1455240_x_at	LOC666185	Similar to CG32602-PA	2.46
1451260_at	Aldh1b1	aldehyde dehydrogenase 1 family, member B1	2.45
1435217_at	LOC666185	Similar to CG32602-PA	2.41
1456808_at		Mus musculus transcribed sequences	2.36
1446187_at		Mus musculus transcribed sequence with moderate similarity to protein pir:S12207 (M.musculus) S12207 hypothetical protein (B2 element) - mouse	2.30
1443272_at	Nope	neighbor of Punc E11	2.28
1456169_at		predicted gene, EG226654	2.28

Probe set	Gene	Gene Title	Fold change
1436503_at		cDNA sequence BC048546	2.27
1420342_at	Gdap10	ganglioside-induced differentiation-associated-protein 10	2.24
1456812_at	Abcd2	ATP-binding cassette, sub-family D (ALD), member 2	2.23
1439149_s_at		predicted gene, ENSMUSG00000056509	2.18
1455037_at	Plxna2	plexin A2	2.17
1440587_at		Transcribed locus	2.14
1418174_at	Dbp	D site albumin promoter binding protein	2.13
1425834_a_at	Gpam	glycerol-3-phosphate acyltransferase, mitochondrial	2.13
1453286_at	Plxna2	plexin A2	2.12
1432997_at		RIKEN cDNA 5830462P14 gene	2.11
1446626_at		DNA segment, Chr 16, human D22S680E, expressed	2.10
1442049_at		Transcribed locus	2.10
1458816_at		Mus musculus transcribed sequences	2.10
1423226_at	Ms4a1	membrane-spanning 4-domains, subfamily A, member 1	2.08
1415894_at	Enpp2	ectonucleotide pyrophosphatase/phosphodiesterase 2	2.07
1451681_at		cDNA sequence BC089597	2.06
1438788_at		DNA segment, Chr 5, Wayne State University 152, expressed	2.06
1417267_s_at	Fkbp11	FK506 binding protein 11	2.06
1453304_s_at	Ly6e	lymphocyte antigen 6 complex, locus E	2.06
1443533_at		Transcribed locus	2.05
1425646_at		cDNA sequence BC016495	2.03
1456648_at		Transcribed locus	2.01
1419146_a_at	Gck	glucokinase	1.99
1440700_a_at	Arhgef18	rho/rac guanine nucleotide exchange factor (GEF) 18	1.99
1421422_at		RIKEN cDNA 5033411D12 gene	1.99
1460546_at	Lgi3	leucine-rich repeat LGI family, member 3	1.96
1423566_a_at	Hsph1	heat shock 105kDa/110kDa protein 1	1.94
1419209_at	Cxcl1	chemokine (C-X-C motif) ligand 1	1.93
1436590_at	Ppp1r3b	protein phosphatase 1, regulatory (inhibitor) subunit 3B	1.93
1420928_at	St6gal1	beta galactoside alpha 2,6 sialyltransferase 1	1.92
1448261_at	Cdh1	cadherin 1	1.92
1425918_at	Egln3	EGL nine homolog 3 (C. elegans)	1.92
1417936_at	Ccl9	chemokine (C-C motif) ligand 9	1.91
1438580_at	Zcchc7	zinc finger, CCHC domain containing 7	1.91
1421830_at	Ak3l1	adenylate kinase 3 alpha-like 1	1.90
1449385_at	Hsd17b6	hydroxysteroid (17-beta) dehydrogenase 6	1.90
1437886_at	Klhl6	kelch-like 6 (Drosophila)	1.90
1438454_at		RIKEN cDNA B430203M17 gene	1.89
1433874_at	Ssh1	slingshot homolog 1 (Drosophila)	1.87
1441326_at	Cp	ceruloplasmin	1.86
1457580_at	Chd8	chromodomain helicase DNA binding protein 8	1.86
1418697_at	Inmt	indolethylamine N-methyltransferase	1.86
1455418_at		Transcribed locus	1.86
1417761_at	Apoa4	apolipoprotein A-IV	1.85
1450341_at	Pcdhb8	protocadherin beta 8	1.85

Probe set	Gene	Gene Title	Fold change
1442097_at		Transcribed locus	1.85
1437466_at	Alcam	activated leukocyte cell adhesion molecule	1.84
1433691_at	Ppp1r3c	protein phosphatase 1, regulatory (inhibitor) subunit 3C	1.84
1448136_at	Enpp2	ectonucleotide pyrophosphatase/phosphodiesterase 2	1.83
1418904_at	Gfpt1	glutamine fructose-6-phosphate transaminase 1	1.83
1418405_at	Hgfac	hepatocyte growth factor activator	1.83
1443172_at	Orc1l	origin recognition complex, subunit 1-like (S.cereviaiae)	1.83
1426302_at	Tmprss4	transmembrane protease, serine 4	1.83
1438245_at		Mus musculus transcribed sequences	1.83
1439537_at		Mus musculus transcribed sequences	1.83
1419193_a_at	Gmfg	glia maturation factor, gamma	1.82
1425993_a_at	Hsph1	heat shock 105kDa/110kDa protein 1	1.82
1441361_at	Mpdz	multiple PDZ domain protein	1.81
1416808_at	Nid1	nidogen 1	1.81
1444458_at		Mus musculus transcribed sequence with moderate similarity to protein pir:S12207 (M.musculus) S12207 hypothetical protein (B2 element) - mouse	1.81
1448898_at	Ccl9	chemokine (C-C motif) ligand 9	1.80
1457644_s_at	Cxcl1	chemokine (C-X-C motif) ligand 1	1.80
1423275_at	Ints6	integrator complex subunit 6	1.80
1432857_at		RIKEN cDNA 6430500D05 gene	1.80
1440327_at	AI195470	expressed sequence AI195470	1.79
1428067_at	Rasl12	RAS-like, family 12	1.79
1447255_at		RIKEN cDNA 2310015A10 gene	1.79
1434283_at		similar to modulator recognition factor 2	1.79
1436767_at	Luc7l2	LUC7-like 2 (S. cerevisiae)	1.78
1429900_at		RIKEN cDNA 5330406M23 gene	1.78
1439861_at	Zfp583	zinc finger protein 583	1.78
1426300_at	Alcam	activated leukocyte cell adhesion molecule	1.77
1456855_at		Mus musculus transcribed sequences	1.77
1439189_at	Fnip2	folliculin interacting protein 2	1.76
1425503_at	Gcnt2	glucosaminyl (N-acetyl) transferase 2, I-branching enzyme	1.75
1435740_at		predicted gene, ENSMUSG00000072684	1.75
1450387_s_at	Ak3l1	adenylate kinase 3 alpha-like 1	1.74
1435917_at	Ociad2	OCIA domain containing 2	1.74
1424968_at		RIKEN cDNA 2210023G05 gene	1.74
1436976_a_at	Yod1	YOD1 OTU deubiquitinating enzyme 1 homologue (S. cerevisiae)	1.74
1427561_a_at	Afm	afamin	1.73
1456857_at		RIKEN cDNA 1500011B03 gene	1.73
1437868_at		cDNA sequence BC023892	1.72
1420123_at	Tcta	T-cell leukemia translocation altered gene	1.72
1424609_a_at		cDNA sequence BC003993	1.71
1428231_at	Cpsf6	cleavage and polyadenylation specific factor 6	1.71
1439161_at	Saps3	SAPS domain family, member 3	1.71
1417154_at	Slc25a14	solute carrier family 25 (mitochondrial carrier, brain), member 14	1.71
1449955_at	Cacna1f	calcium channel, voltage-dependent, alpha 1F subunit	1.70

Probe set	Gene	Gene Title	Fold change
1428230_at	Prkd3	protein kinase D3	1.70
1443460_at		Hypothetical protein LOC100043131	1.69
1429796_at	Kalrn	kalirin, RhoGEF kinase	1.69
1437983_at	Sall1	sal-like 1 (Drosophila)	1.69
1425394_at		cDNA sequence BC023105	1.68
1428229_at	Prkd3	protein kinase D3	1.68
1439625_at		Transcribed locus, moderately similar to XP_001064639.1	1.68
1420772_a_at	Tsc22d3	TSC22 domain family 3	1.68
1445322_x_at		Mus musculus transcribed sequence with weak similarity to protein sp:Q9H321 (H.sapiens) VCXC_HUMAN VCX-C protein (Variably charged protein X-C)	1.68
1456026_at	Nhlrc3	NHL repeat containing 3	1.67
1416258_at	Tk1	thymidine kinase 1	1.67
1437467_at	Alcam	activated leukocyte cell adhesion molecule	1.66
1452678_a_at	Ccbl1	cysteine conjugate-beta lyase 1	1.66
1425631_at	Ppp1r3c	protein phosphatase 1, regulatory (inhibitor) subunit 3C	1.66
1444273_at		expressed sequence AW555355	1.65
1418265_s_at	Irf2	interferon regulatory factor 2	1.65
1447951_at		Transcribed locus	1.65
1427440_a_at	Afm	afamin	1.64
1428151_x_at	Ccbl1	cysteine conjugate-beta lyase 1	1.64
1450084_s_at	Ivns1abp	influenza virus NS1A binding protein	1.64
1443078_at		RIKEN cDNA 6030439D06 gene	1.64
1426301_at	Alcam	activated leukocyte cell adhesion molecule	1.63
1427371_at	Abca8a	ATP-binding cassette, sub-family A (ABC1), member 8a	1.63
1427137_at	Ces5	carboxylesterase 5	1.63
1438348_x_at	Ccbl1	cysteine conjugate-beta lyase 1	1.63
1448185_at	Herpud1	homocysteine-inducible, endoplasmic reticulum stress-inducible, ubiquitin-like domain member 1	1.63
1425127_at	Hsd3b2	hydroxy-delta-5-steroid dehydrogenase, 3 beta- and steroid delta-isomerase 2	1.63
1425718_a_at	Ivns1abp	influenza virus NS1A binding protein	1.63
1418189_s_at	Malat1	metastasis associated lung adenocarcinoma transcript 1 (non-coding RNA)	1.63
1438188_x_at	Slc25a29	solute carrier family 25 (mitochondrial carrier, palmitoylcarnitine transporter), member 29	1.63
1435626_a_at	Herpud1	homocysteine-inducible, endoplasmic reticulum stress-inducible, ubiquitin-like domain member 1	1.62
1456216_at		Mus musculus transcribed sequence with weak similarity to protein pir:I58401 (M.musculus) I58401 protein-tyrosine kinase (EC 2.7.1.112) JAK3 - mouse	1.61
1452353_at	Gpr155	G protein-coupled receptor 155	1.60
1438187_at	Slc25a29	solute carrier family 25 (mitochondrial carrier, palmitoylcarnitine transporter), member 29	1.60
1455282_x_at	Alas1	aminolevulinic acid synthase 1	1.59
1428393_at	Nrn1	neuritin 1	1.59
1452767_at	Rrbp1	ribosome binding protein 1	1.59
1425281_a_at	Tsc22d3	TSC22 domain family 3	1.59
1428585_at	Actn1	actinin, alpha 1	1.58
1460256_at	Car3	carbonic anhydrase 3	1.57

Probe set	Gene	Gene Title	Fold change
1424210_at	Erlin1	ER lipid raft associated 1	1.57
1423319_at	Hhex	hematopoietically expressed homeobox	1.57
1420621_a_at	App	amyloid beta (A4) precursor protein	1.56
1431803_at	Cyp2d13	cytochrome P450, family 2, subfamily d, polypeptide 13	1.56
1460249_at	Lnx2	ligand of numb-protein X 2	1.56
1418979_at	Akr1c14	aldo-keto reductase family 1, member C14	1.55
1440899_at	Fmo5	flavin containing monooxygenase 5	1.55
1451969_s_at	Parp3	poly (ADP-ribose) polymerase family, member 3	1.54
1445248_at		gb:BG074862 /DB_XREF=gi:12557431 /DB_XREF=H3140C08-3	1.54
1421840_at	Abca1	ATP-binding cassette, sub-family A (ABC1), member 1	1.53
1417339_a_at	Dynll1	dynein light chain LC8-type 1	1.53
1451668_at		RIKEN cDNA C530043G21 gene	1.53
1448484_at	Amd-ps3	S-adenosylmethionine decarboxylase 1	1.53
1416563_at	Ctps	cytidine 5'-triphosphate synthase	1.52
1439476_at	Dsg2	desmoglein 2	1.52
1416188_at	Gm2a	GM2 ganglioside activator protein	1.52
1427287_s_at	Itpr2	inositol 1,4,5-triphosphate receptor 2	1.52
1447774_x_at		RIKEN cDNA 5730469M10 gene	1.52
1424028_at		RIKEN cDNA 5830457O10 gene	1.52
1456381_x_at	Mcl1	myeloid cell leukemia sequence 1	1.51
1425515_at	Pik3r1	phosphatidylinositol 3-kinase, regulatory subunit, polypeptide 1 (p85 alpha)	1.51
1418401_a_at	Dusp16	dual specificity phosphatase 16	1.50
1438167_x_at	Flcn	Folliculin	1.50
1418447_at	Golga5	golgi autoantigen, golgin subfamily a, 5	1.50
1423028_at	Ifna2	interferon alpha 2	1.50
1436240_at		Transcribed locus	1.50
1436162_at		RIKEN cDNA C730048C13 gene	1.49
1428112_at	Armet	arginine-rich, mutated in early stage tumors	1.48
1424996_at	Cflar	CASP8 and FADD-like apoptosis regulator	1.48
1427377_x_at	Hsd3b3	hydroxy-delta-5-steroid dehydrogenase, 3 beta- and steroid delta-isomerase 3	1.48
1450919_at	Mpp1	membrane protein, palmitoylated	1.48
1416880_at	Mcl1	myeloid cell leukemia sequence 1	1.48
1439560_x_at		predicted gene, EG432995	1.48
1455500_at		ring finger protein 213	1.48
1460694_s_at	Svil	supervillin	1.48
1456437_x_at	C1r	complement component 1, r subcomponent	1.47
1452896_at	Gtl3	gene trap locus 3	1.47
1460606_at	Hsd17b13	hydroxysteroid (17-beta) dehydrogenase 13	1.47
1426397_at	Tgfbr2	transforming growth factor, beta receptor II	1.47
1455396_at	Atp8b1	ATPase, class I, type 8B, member 1	1.45
1453263_at	Mak10	MAK10 homolog, amino-acid N-acetyltransferase subunit, (S. cerevisiae)	1.45
1426752_at	Phf17	PHD finger protein 17	1.45
1451210_at	Ppap2c	phosphatidic acid phosphatase type 2c	1.45
1452944_at	Afmid	arylformamidase	1.44
1416755_at	Dnajb1	DnaJ (Hsp40) homolog, subfamily B, member 1	1.44

Probe set	Gene	Gene Title	Fold change
1452416_at	Il6ra	interleukin 6 receptor, alpha	1.44
1419185_a_at	Mlxipl	MLX interacting protein-like	1.44
1449027_at	Rhou	ras homolog gene family, member U	1.44
1442510_at		similar to LOC360919 protein	1.44
1424374_at	Gimap4	GTPase, IMAP family member 4	1.43
1455597_at	Map3k2	mitogen-activated protein kinase kinase kinase 2	1.43
1434102_at	Nfib	nuclear factor I/B	1.43
1438714_at		Transcribed locus	1.43
1449740_s_at	Dsg2	desmoglein 2	1.42
1426903_at	Fndc3a	fibronectin type III domain containing 3a	1.42
1428681_at	Gm608	gene model 608, (NCBI)	1.42
1420420_at	Hao1	hydroxyacid oxidase 1, liver	1.42
1419043_a_at	Iigp1	interferon inducible GTPase 1	1.42
1423725_at	Pls3	plastin 3 (T-isoform)	1.42
1427464_s_at	Hspa5	heat shock protein 5	1.41
1449028_at	Rhou	ras homolog gene family, member U	1.41
1437308_s_at	F2r	coagulation factor II (thrombin) receptor	1.40
1435357_at		DNA segment, Chr 4, Wayne State University 53, expressed	1.40
1426645_at	Hsp90aa1	heat shock protein 90, alpha (cytosolic), class A member 1	1.40
1428872_at		RIKEN cDNA 4121402D02 gene	1.40
1424041_s_at	C1s	complement component 1, s subcomponent	1.39
1456125_a_at	Dynll1	dynein light chain LC8-type 1	1.39
1449221_a_at	Rrbp1	ribosome binding protein 1	1.39
1418069_at	Apoc2	apolipoprotein C-II	1.38
1456609_at	Camk2n1	calcium/calmodulin-dependent protein kinase II inhibitor 1	1.38
1450852_s_at	F2r	coagulation factor II (thrombin) receptor	1.38
1419462_s_at	Gtl3	gene trap locus 3	1.38
1420913_at	Slco2a1	solute carrier organic anion transporter family, member 2a1	1.38
1420723_at	Vnn3	vanin 3	1.38
1415961_at	Itm2c	integral membrane protein 2C	1.37
1451421_a_at	Rogdi	rogdi homolog (Drosophila)	1.37
1416288_at	Dnaja1	DnaJ (Hsp40) homolog, subfamily A, member 1	1.36
1416064_a_at	Hspa5	heat shock protein 5	1.35
1427442_a_at	App	amyloid beta (A4) precursor protein	1.33
1452734_at	Gm1604	ribonuclease T2B	1.33
1426123_a_at	Rrbp1	ribosome binding protein 1	1.32
1425387_at	Akr1c20	aldo-keto reductase family 1, member C20	1.31
1420622_a_at	Hspa8	heat shock protein 8	1.27
